# Males of a sexually cannibalistic spider chemically assess relative female quality

**DOI:** 10.1186/s12862-020-01657-w

**Published:** 2020-07-23

**Authors:** Anna-Lena Cory, Jutta M. Schneider

**Affiliations:** Institute of Zoology, Universität Hamburg, Hamburg, Germany

**Keywords:** Male mate choice, Monogyny, Sampling strategies, Sex pheromones, Sexual cannibalism, Sperm competition

## Abstract

**Background:**

Mate choice is a taxonomically wide-spread phenomenon, mostly exerted by females although male mate choice occurs as well. While costs and benefits of choosiness have been well studied, the underlying mechanisms are largely unclear. Different models exist, namely best-of-n or threshold rules, based on sequential or simultaneous sampling, which differ in the required cognitive demands. We applied an experimental approach to shed light on the underlying mechanisms of male mate choice in the sexually cannibalistic spider *Argiope bruennichi.* Males are limited to two copulations and preferentially monopolise large females, while they may leave smaller females after a single copulation and resume mate search. Here, we utilised significant size-differences between females from Northern and Southern populations and presented males with three different-sized females that were matched for origin: all three females originated either from the same Northern European population as the males or from Southern populations where the smallest female was about the same size as the largest Northern female. This allowed testing the hypothesis that males base their mating tactic on a fixed local size threshold. We predicted Northern males to be choosy among Northern females, but to accept all Southern females since they would all be above that threshold.

**Results:**

Males copulated with the first female they encountered, which was independent of her body size. Regardless of the females’ origins, males chose a monogynous tactic with the largest female in the trio, while they left the smallest female after one copulation. The same pattern applied to Southern females even though the smallest females in the trio were of a similar size as monopolised Northern females. Since males have poor eyesight and did not actively sample all females, they likely have gained information about relative size differences between females based on volatile chemical cues only.

**Conclusions:**

Our findings suggest that male *A. bruennichi* can assess relative differences in mate quality and adjust their mating tactic to the prevailing conditions (Northern vs. Southern). We reject the presence of a locally-adapted fixed threshold and argue that our results are best explained by an adjustable threshold that was raised under Southern conditions.

## Background

Mate choice decisions occur in a broad range of animal taxa and several models try to explain the underlying rules and mechanisms of the decision process, but only a few have yet been tested [1]. Generally, the evolution of choosiness requires the opportunity and the ability of a comparative assessment of potential mates [2, 3]. However, the ability to compare mates and make a decision may demand different degrees of cognitive investment depending on the underlying mate choice rule.

Cognitive demands are the highest for a comparative assessment following the best-of-n strategy which entails that individuals simultaneously or sequentially compare and choose among a group of potential mates [3–6]. A sequential assessment demands extended memory and virtual comparisons. Therefore, the latter should only evolve under very specific conditions such as a short latency between encounters [6, 7]. In comparison, a threshold rule is less costly and requires no memory and is more likely to evolve even in small-brained animals [6, 8, 9]. In line with this, several studies investigating sequential or simultaneous mate choice decisions in invertebrates, mostly crustaceans and insects, signify that females compare male quality to a fixed standard [10–12]. However, in environments where variation in mate quality is inconsistent over space or time, a fixed standard in mate quality may be too high or low and lead to fitness losses due to suboptimal choices. Interestingly, some studies found that a threshold is not necessarily a rigid condition; it can also be lowered or raised allowing adjustments to prevailing mate quality conditions [10, 13–16]. For instance, the threshold to accept a mating partner can be adjusted to the quality of the previous one (one-step decision rule; 13, 15). Other studies found that a threshold may be lowered over time if an animal only encounters potential mating partners below the threshold [10, 14]. However, as with the best-of-n strategy, an adjustable threshold requires a comparative assessment by which either the previous conditions or an internal standard is compared to the preveiling conditions.

Models of comparative mate choice rules assume an initial information sampling phase in which at least two potential mates are compared [3, 6]. While most mate choice experiments are indeed binary, sampling becomes more complex and costly with increasing options [1, 5, 6, 17]. The number of mates being assessed can be observed easily in animals where information is gathered through direct inspection visits that can be followed and counted [18, 19]. However, acoustic or chemical signals can be perceived from a distance and potentially compared without changing position [20, 21]. To evaluate the sampling, we need to know what the animal can detect and process and up to which distance information is perceived. Unfortunately, such information is only available for a limited number of species as for example for acoustic sexual signalling in crickets or frogs (e. g [22–24].).

Here we investigate how male spiders likely having poor vision, but plenty of chemoreceptive sensilla [25, 26] decide on their mating tactic when confronted with several females of different quality. In mating systems with traditional sex roles, females are generally the choosy sex while male mate choice evolves under conditions of high male mating effort or paternal investment and considerable differences in quality between females [2, 27–29]. Sexually selected female traits are usually related to fecundity and mating status, particularly when sperm precedence patterns are present [30–33].

**Fig. 1:**
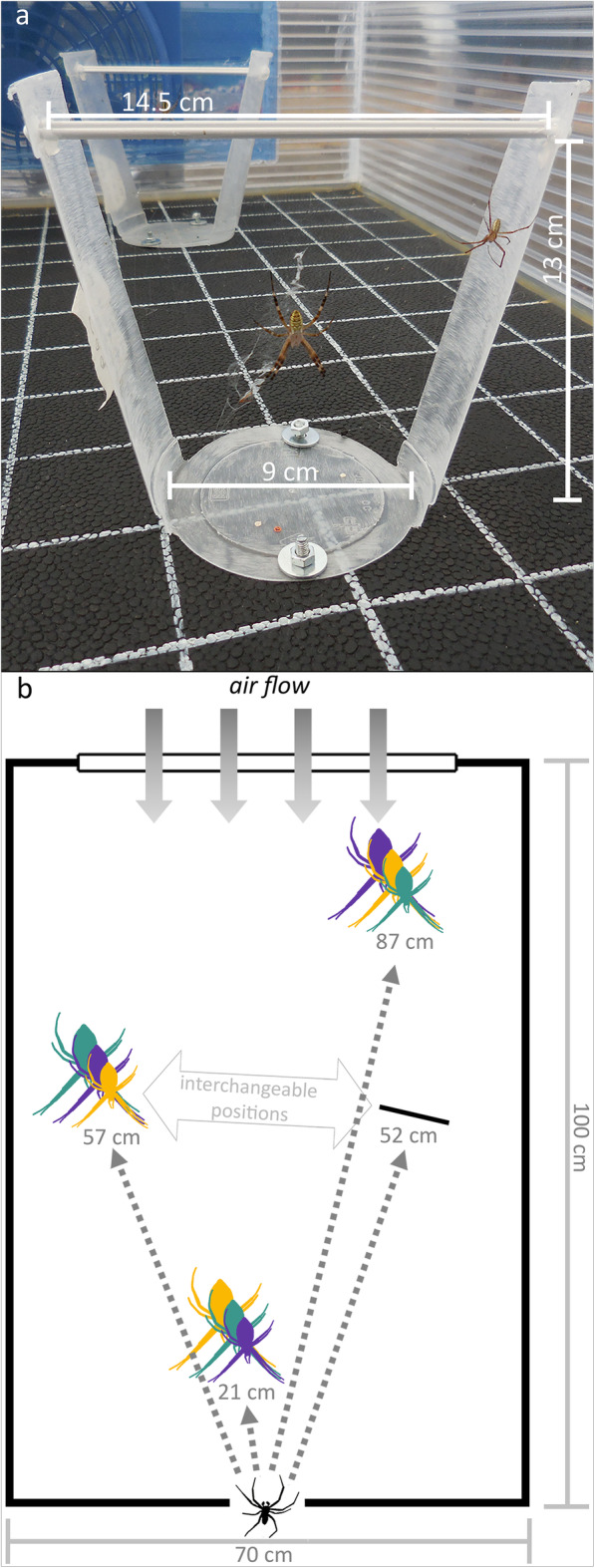
Design of test arenas. **a** Photograph of *Argiope bruennichi* female (centre) in her web with a male (right) inside a test frame. The test frames were custom-made and allowed for stationing females on fixed positions within the arena. The photograph was taken by Anna-Lena Cory. **b** Scheme of the test arena. Males could choose between three females that were matched for origin (Southern or Northern) and differed in size and distance from the male’s starting point (lowest spider silhouette), whereby there were two options for the middle-distant position. Three of six possible distribution patterns are illustrated by different colours of female silhouettes (from close to far distant: dark purple = small-medium-large, blue-green = medium-large-small, amber = large-small-medium). Note that the scheme does not depict that males were exposed to females from either Northern Europe or Southern Europe only. Adobe Photoshop was used to create the scheme. A licence is held by the Universität Hamburg

In web-spiders, males are the searching sex, and they find females through volatile sex pheromones emitted by the female and the web [34]. While the chemical structures of pheromones have been analysed in a few spider species, nearly nothing is known about the information contained in these volatile semiochemicals [35, 36]. Components on the silk of females, however, have been shown to contain information on mating status, diet and condition in several *Argiope* species [37–41] and in the black widow *Latrodectus hesperus* where males are less responsive to contact pheromones of starving females than of sated ones [42]. This differentiation is adaptive because hungry females pose a higher risk for males to get cannibalised during courtship [42]. Particularly in species in which males are regularly cannibalised by females, males could benefit from chemical information about female quality that they receive from a distance before entering female webs. Females of the sexually cannibalistic orb-web spider *Argiope bruennichi* (Fig. 1a), for instance, show a high variation in body size, of which fecundity is a direct function [43–45]. Females attack every male during copulation and often kill males during their first copulation [46]. Males damage their paired genitalia during copulation and can maximally copulate twice, once with each mating organ [47]. Hence, dying after one copulation reduces male mating rates by half [46]. Males that survive their first copulation can then decide whether to mate a second time with the same female (monogyny) or to leave and inseminate a second female (bigyny). Accordingly, males are monogynous either because they are cannibalised by the female already after their first copulation, or because they survive and choose to copulate with the same female again. Survival of the first copulation is only possible if the male jumps off before 10 s have passed and we consider death after such a short copulation a consequence of an unsuccessful escape attempt [48]. Chances of survival rapidly approach zero after this, such that a single long copulation may mean that a male sacrificed his future reproduction in favour of transferring more sperm during his first copulation [48]. In any case, these males leave one genital opening free for a potential future sire. However, not all females remate, as one copulation is sufficient to fertilise all her eggs [49].

Males that copulate twice with the same female minimise the risk of sperm competition because both female genital openings are securely plugged, and further copulations are unlikely to result in paternity (monopolisation; 46, 47). On the other hand, bigynous males can potentially sire more offspring if they successfully mate with two virgin females and particularly if these females do not re-mate. Accordingly, males have the option to allocate their mating effort strategically. A dynamic game-theoretical model based on data from *A. bruennichi* confirmed that male mate choice evolves under realistic conditions [50]. However, it is hardly understood how males find their first female to mate with and if the availability of alternative mating options influences their decisions. Simultaneous mate choice experiments showed that males more likely visited and mated with virgin females, while body size and condition only played a role for males that survived the first copulation [51, 52]. Field observations revealed that surviving males chose a monopolising tactic preferentially with large and fecund females [52]. How males recognise large females (e. g. chemical cues or tactical cues in the web) and which sampling strategy underlies this size-dependent mating strategy still waits to be tested.

Previous studies suggest that males rather use a threshold rule than a best-of-n strategy [51, 53, 54]. Under best-of-n strategy, males should either sample several females before they mate or they should immediately visit the largest female, if they are able to distinguish females of different body size via chemical cues. However, in several previous studies, males showed neither of these mate searching patterns [51, 54]. In contrast, males adjusted their mating strategy (monogyny, bigyny) to female body size even though no alternative mating options were available; an observation that complies with a threshold rule [53]. As female body size is highly variable [53], it might be beneficial if males are able to recognise the overall quality of potential mates nearby and adjust their threshold accordingly. Males are very sensitive to female pheromones and may assess the overall female quality in a patch from a distance. Therefore, our goal was to experimentally test whether males indeed follow a threshold rule and whether the threshold is fixed or adjustable.

We explored male decision rules, by offering naïve *A. bruennichi* males a choice between three virgin females of different size (small, medium, large) in large test arenas (Fig. 1b). To distinguish between a fixed threshold and an adjustable threshold, we made use of geographical differences in female size and fecundity: females from populations in Southern Europe are significantly larger than females from populations in Northern Europe [44]. We exposed males from Northern populations to three females that either came from the Northern (treatment 1) or Southern populations (treatment 2). As a consequence of their different origins, the size distribution of the three females differed significantly between the two treatments. We presented the females at different distances relative to the male starting position (Fig. 1b) with the aim to mimic a natural situation as well as the conditions in previous studies [51, 52, 54]. The distribution of different sized females among these positions was randomised so that distance and female size could be analysed independently. During trials, we monitored which female was encountered first, the duration of the first copulation, the occurrence of sexual cannibalism and the chosen mating tactic. Under the ***adjustable threshold hypothesis***, males should always decide on the largest female regardless of the absolute size differences between Northern and Southern females. The ***fixed threshold hypothesis*** on the other hand predicts that males should not be selective when encountering Southern females since their body sizes would all be above the local Northern threshold.

## Results

### Choosing the first female

Before moving into the arena, males raised their front legs where many sensory hairs are located [26]. Males differed in their search patterns. While some males walked to female webs without detours, others went back and forth or initially followed the walls and found their way to the webs from there. Half of the males took less than 2 min (interquartile range: 1–16 min) to arrive at their first web. All but two males (N_total_ = 54) copulated with the female that they encountered first. However, which female they visited first did not depend on distance or female size class (trials with Southern and Northern females combined). The first encounter was not more likely with the nearest female (close = 33.3%; medium = 24.1%, far = 42.6%; Chi^2^ test: *N* = 54, Chi^2^ = 1.444, df = 2, *p* = 0.49) nor with the largest female in the arena (small = 25.9%, medium-sized = 46.3%; large = 27.8%; Chi^2^ test: *N* = 54, Chi^2^ = 2.11, df = 2, *p* = 0.35). Moreover, we found no indication that males rather passed the closest female if she was small (logistic regression; response variable: mated with closest female (yes/no), explanatory variable: mated with small female (yes/no); *N* = 54, ΔAICc = 0.75, weight = 0.965).

### Copulation duration and occurrence of sexual cannibalism (monogyny after a single copulation)

Males copulated for a median duration of 6.2 s (interquartile range: 5.2–8.4 s). A two-way ANOVA (full model: *N* = 52, F_48,3_ = 0.48, *p* = 0.701) revealed that neither female origin (*N* = 52, F_48,1_ = 0.63, *p* = 0.43) nor female size class (*N* = 52, F_48,2_ = 0.40, *p* = 0.67) influenced the duration of copulation. However, corroborating earlier findings, cannibalised males copulated for longer than males that survived (Mann-Whitney U test: *N* = 54, W = 490, *p* = 0.02). Five males copulated longer than 10 s, and only one of these males (copulation duration: 10.67 s) survived. These longer copulations do not appear to relate to female size class (2 small, 2 medium-sized and 1 large female) or female origin (2 Northern, 3 Southern females). To test whether female size or origin predicted sexual cannibalism, we conducted a model selection analysis and compared the AICc values of different models (see Methods, Table 1). Although Southern females cannibalised more males (70.3%) compared to Northern females (44.4%), the model with female origin as predictor was not better than the null model (Table 1).

**Table 1:** Summary of model selection analysis to reveal influential effects on the cannibalism rate

Model variables	K	AICc	ΔAICc	weight
♀ origin	2	74.1	0.00	0.366
♀origin + ♀ size class	4	75.1	0.94	0.228
♀ size classes	3	75.2	1.02	0.219
**Null model**	**1**	**75.7**	**1.60**	**0.164**
♀ origin + ♀ size class + ♀ origin:♀ size class	7	82.3	5.57	0.023

*For each model, we used a binary logistic regression (N = 54) and listed them in descending order of their Akaike information criterion corrected for small samples (AICc). The model with the lowest AICc value was generally the most plausible model. We suggested other models as equally plausible if their AICc value differentiated less than 2 units from the most plausible model (grey background). From these models, we selected the model with the fewest parameters (K) (bold letters).****Δ****AICc = AICc difference to the most plausible model; weights = AICc weights*

### Monogyny (=monopolisation) or bigyny

We monitored whether males stayed and re-mated with their first mate (monogyny) or whether males left the first mate up to 1 h after they escaped from their first copulation. We discarded the 31 males that died during their first copulation, since we cannot unambiguously define this as a male decision. In total, 23 males survived the first copulation, of which 13 males left their first mate. Seven of them moved into the web of a second female that was larger even if the first female was in the medium size class. Within the 1 h observation time, only two males copulated with the second female that in both cases belonged to the size class “large”. We classified the remaining males as “intended bigynous” if they did not return to the first female within an hour and if they showed a clear distance to the first mating partner without any contact (directly or by silk threads) to the female’s web or the test frame.

**Fig. 2:**
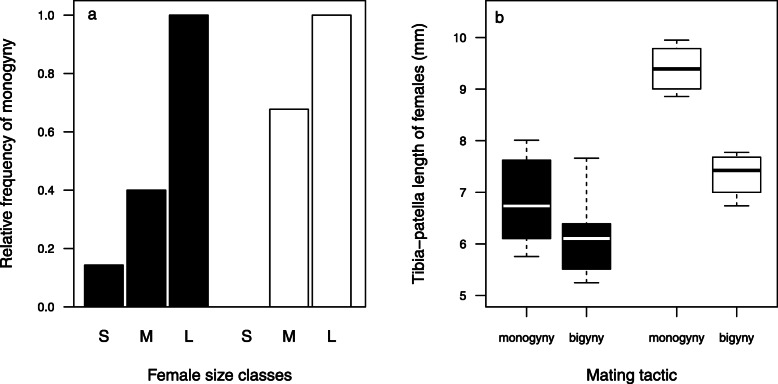
Effects of female body size on male mating tactics. The plots (Fig. **a**, **b**) depict the effects of body size of Northern (black) and Southern (white) females on mating tactics in males that survived the first copulation. **a** Relative frequency of monogyny dependent on female size classes (S = small, M = medium-sized, L = large). **b** Boxplots presenting the body size (leg length) of first female mates chosen from monogynous and bigynous males

Among the 23 males that survived their first copulation, ten (43.5%) mated a second time with their first mate and thereby monopolised paternity. The probability of monogyny (see Methods, Table 2) was best explained by the model including only female size class as predictor (Table 2). With increasing female size class, males were more likely monogynous (Table 3, Fig. 2b). Indeed, all females from the size class “large” were monopolised (*N* = 5), but nine out of ten “small” females were left after the first copulation (Fig. 2b). Within the group of medium-sized females, four of eight females were monopolized (Fig. 2a). The relationship between the likelihood of monogyny and female size was still present when we dropped the size classes and used the leg length as an index for the body size of females instead (logistic regression: best model includes female size, *N* = 23, ΔAICc = 6.62, weight = 0.965). The use of female size as a continuous variable further revealed that males left Southern females that were approximately as large as Northern females that males monopolised (Fig. 2b).

**Table 2:** Summary of model selection analysis to reveal influential effects on mating tactics (mono- versus bigyny) of males that survived the first copulation

Model variables	K	AICc	ΔAICc	weight
**♀ size class**	**3**	**25.8**	**0.00**	**0.801**
♀origin + ♀ size class	4	28.9	3.06	0.173
Null model	1	33.7	7.86	0.016
♀ origin	2	35.9	10.06	0.005
♀ origin + ♀ size class + ♀ origin:♀ size class	7	35.9	10.19	0.005

*We used a Firth-corrected binary logistic regression (N = 23) for each model and listed them in descending order of their Akaike information criterion corrected for small samples (AICc). The model with the lowest AICc value was generally the most plausible model. We suggested other models as equally plausible if their AICc value differentiated less than 2 units from the most plausible model (grey background). From these models, we selected the model with the fewest parameters (K) (bold letters).****Δ****AICc = AICc difference to the most plausible model; weights = AICc weights*

**Table 3:** Summary of results of the most plausible model to predict male mating tactics

Female size class	Estimates ± SE	Z	P
Intercept (small ♀♀)	−1.85 ± 0.92	−2.00	0.045*
Medium-sized ♀♀	1.85 ± 1.16	1.60	0.111
Large ♀♀	4.27 ± 1.88	2.28	0.025*

*In a Firth-corrected binary logistic regression (N = 23), we tested the effects of female size class on the probability of monogyny in males that survived the first copulation. The estimates and standard errors (SE) of the estimates are Firth bias-corrected and logit-transformed. Asterisks indicate significant values*

### Reproductive success of monopolising versus bigynous males

The reproductive success of monogynous males that copulate twice can be estimated by the fecundity of the female they monopolised. Reproductive success of bigynous males will also depend on the re-mating probability of their first female and on their chances to find a 2nd female plus the fecundity of that one. Comparing reproductive success between those two strategies is therefore difficult unless we know these probabilities. Here we restrict the comparison to what mono- and bigynous males gained from the first egg sacs of the females they mated with.

We weighed egg-sacs from those females used in the choice trials but we did not open cocoons to count the eggs. However, since egg number is highly correlated with egg-sac weight, we estimated egg-numbers of first egg-sacs by using a calibration curve derived from egg-sacs of other Northern females of the same cohorts (linear regression: y = 1.3029x + 74.9097, *N* = 66, F_1,64_ = 127.06, *p* < 0.0001, adjusted R^2^ = 0.6598). Using the regression of egg-sac weight on egg number, monogynous males fathered an average of 331.2 ± 69.3 eggs. Bigynous males gained on average 246.9 ± 53.4 eggs from the 1st egg-sac of their female, which is a statistically significant difference (two sample t-test: *N* = 18, t = − 2.91, *p* = 0.0102). The lower reproductive success with 1st females underscores that males opt for bigyny if their first mate is small and less fecund than others.

Female leg length correlated positively with the estimated number of eggs contained in the first egg sac (Spearman rank correlation: *N* = 38, rho = 0.938, *p* < 0.0001) and Southern females produced significantly more eggs than Northern females (Mann-Whitney U test: N = 38, W = 49, p < 0.0001). Hence, males gained a good estimate for expected reproductive success from the body size of a female.

## Discussion

In this study, we tested mating decisions of *Argiope bruennichi* males from Northern European populations by offering them three females of different sizes that either originated from the same Northern population or that all came from a Southern population where females grow much larger. The smallest Southern females were of a comparable size to the largest Northern females. Regardless of the absolute sizes of offered females, males adopted a monogynous mating tactic if the first female they encountered was relatively large and they were bigynous if they had encountered a relatively small female first. This was adaptive because females of monogynous males produced on average 25% more eggs than the first females of bigynous males. While relative size was important, absolute female sizes played no role, as bigynous males left the relatively smallest Southern females even though they were as large as the largest Northern females. These findings are not consistent with the ***fixed threshold hypothesis*** but lend support to the ***adjustable threshold hypothesis***. We conclude that males selected their mate choice tactic based on information they must have gained about the relative quality of females nearby (patch quality). This occured even though males did not visit all females within a patch and it was also independent of whether larger females sat up- or downwind. Curiously, males did not use this information to find their first mate. In summary, our results suggest that during mate search males do not perceive or consider female size. However, once they are in the web of their 1st mate, that female’s relative size determines male mating tactic.

This study experimentally confirms earlier observations of male strategic mate choice decisions. The results are consistent with a trading-up mate choice mechanism based on the assessment of relative female size differences [52]. Our findings suggest that males acquire and use information about the quality of females in the vicinity and adjust their behaviour without visiting the webs of females. Unless males relied on their eye-sight which is regarded although not tested as being rather poor, they most probably accomplished that by using volatile chemical cues or sex pheromones [25, 34, 55]. Several studies on spiders have shown that chemical cues and sex pheromones offer males information about female quality traits [21, 37, 40, 56–58]. In *A. bruennichi*, at least one sex pheromone was identified and is exclusive to virgin females [59]. If males use pheromones to distinguish females of different quality, either the quality or quantity of pheromones must differ between females of different sizes and fecundity. There is evidence in moths that the quantity of pheromones correlates with female quality [60, 61]. Similarly, sex pheromone titres of male *Nasonia vitripennis* were found to correlate with sperm numbers and females were found to use pheromone titres to identify sperm-limited males [62]. Whether pheromone emission in spiders qualifies as an honest signal of quality and is used by males to choose their mating tactic, still waits to be tested.

Our data refute the existence of a fixed threshold rule for mate choice because males monopolised Northern females that had a similar body size as Southern females that were left by males. Instead our results are consistent with an adjustable threshold, which can be lowered or raised if the prevailing conditions deviate from the internal standard. In this study, males might have raised their threshold to accept females of a certain quality when they were exposed to chemical cues of Southern females whose quality was higher than the quality Northern males usually experienced. As underlying mechanism, males might assess the overall mate quality conditions within the area of perception and equalise the internal standard against the actual mate quality conditions in a patch. This explanation is in agreement with a study by Reid and Stamps [16] who tested sampling tactics in female pine engravers (*Ips pini*). Here, male quality differed between patches visited by females and dependent on patch quality, females lowered or raised their threshold [16].

Corroborating previous results, males mated with the first female they encountered regardless of her body size. This suggests that males did not use chemcial cues to assess the quality of specific females and to apply this information to select the best mate for their first choice. Perhaps our set-up mixed the chemical cues of all females and prevented an individual distinction of females. However, laboratory and field tests consistently revealed that *A. bruennichi* males never rejected a first female based on her quality [51, 54]. Rather, males appeared to search randomly and mate with any female they happen to encounter first. Studies on other taxa (e. g. crustaceans, insects and fish) also found that males never rejected the first female, but instead adjusted the investment in a second female depending on the quality of the first one [63–65]. Thus, after they have secured a first mating, males seem to use a low cost sampling strategy that allow for some more flexibility than a fixed threshold would. Flexible mechanisms may be particularly adaptive if males are exposed to unpredictable environments because a high degree of uncertainty will increase the advantages of flexible responses to information about female quality [66]. However, information processing and flexibility in mate choice are also costly and constrained by sensory and neuronal capacities [67]. Moreover, in species where males have a high mating investment, the costs can rapidly exceed the potential benefits of cognitive demanding mate choice mechanisms. A simple mechanism such as an adjustable threshold keeps the costs low such that the equation more likely tilts towards a fitness benefit.

Although our findings are well explained by the hypothesis of an adjustable threshold, we cannot exclude that males evolved yet another mechanism that also allows them to assess and compare the quality of specific females, but with lower cognitive demands than needed for the best-of-n strategy. For instance, female decorated crickets (*Gryllodes sigillatus*) avoid re-mating with the same male by marking mates with their own chemical signature [68]. Thereby, females can compare their own chemical profile with that of a male and thereby detect previous mating partners without learning and remembering a template [68]. A similar mechanism may explain that males only applied a comparative mate choice strategy after securing the first copulation. Males may have to be close to a female to detect whether another female in the vicinity sends a different or a comparatively stronger signal than the present female so that their sensory system would be stimulated despite sitting in a pheromone cloud. This means only relatively short distances between signalling females would enable the male to compare the chemical profile of his mate with that of another signalling female, a prediction that could be tested in future studies. To date, we have no information over which distances our spider males can perceive sex pheromones. A recent field study on *Latrodectus hesperus* found that males perceive female pheromones tens of meters away if wind conditions are favourable [20]. Transmission of chemical or acoustic signals over long distances can be disturbed by many environmental factors (e. g. noise, pollutants, other signalers), which may make a direct comparison of mating partners difficult [22, 69, 70]. Deb and Balakrishnan [22] estimated the active spaces of calling male tree crickets (*Oecanthus henryi*) and found that most females would only hear calls of one male at a time and, hence, would be unable to compare males simultaneously. More research on olfaction of spiders is needed to elucidate how perception is related to behaviour. The increasing number of male choice studies in spiders call for in depth investigations of the mechanisms underlying pheromone production and emission in females as well as chemical and tactile perception mechanisms underlying male decision-making.

So far, we discussed monogyny as a strategy to monopolise large females by plugging both female openings. However, less than 50% of the males survive to accomplish two copulations. Alternatively, males may invest in a single, long copulation and allow the female to cannibalise them during this first copulation. However, in this study, comparisons of copulation duration with small and large females do not indicate that males sacrificed themselves to large females. Also, males did not copulate longer with Southern females, although the cannibalism rate was higher than with Northern females. If anything, our findings indicate that a larger body size gives females an advantage in catching and cannibalising males.

## Conclusions

In conclusion, males of *A. bruennichi* are choosy as they are able to collect reliable information about mate quality and adjust their mating strategies accordingly. Male mate choice has received comparatively little attention but may be more common than appreciated to date. As exemplified in our study system, male choice may not be obvious at first sight, when for example only choices of first mates are considered. We advocate intensifying the search for further examples of sexual selection through mate choice particularly in systems where male mating investment is high.

Our results indicate that males can acquire such information even from a distance and use it to adapt their choice criteria to prevailing conditions. Such adaptations are particularly beneficial if female encounters are dangerous. We can reject the hypothesis of a fixed threshold to adjust mating strategies. Instead, we suggest that the decision rules for applying mating strategies underlie a comparative assessment, most likely resulting in an adjustment of an internal threshold. We propose that future studies should examine the mechanisms behind size-dependent pheromone emission and link the mechanism to the decision-making process in males.

## Methods

### Collection and maintenance

We collected juvenile *Argiope bruennichi* SCOPOLI 1772 from six locations in Southern France (Carcassonne and its surroundings) and nine locations in Northern Germany (Hamburg and its surroundings) between 1st until 22nd June 2014. The collection of these spiders required no permits. Spiders were raised under laboratory conditions with a photoperiod of 16:8 LD and an ambient temperature of ca. 22 °C. They were held in cups of 250, 500 or 1000 ml size depending on the spider’s size. Each week, we provided water on five to six days and fed them twice with ca. 20 *Drosophila* spp. or three *Calliphora* spp. Very small individuals received three to five *Drosophila melanogaster* three times a week. The standardized diet with flies raised on an enriched medium ensured that the females did not show diet related differences in their chemical profile [37, 38, 71]. We checked at least six days a week for sexually mature males and females which can easily be determined by inspection of the genitalia [72].

### Measurements

We took the weight of each female directly after the final moult and before they fed as an adult by using a calibrated scale (Mettler Toledo AB54-S; accuracy 0.1 mg). To avoid stress and harm to the animals, we measured the tibia-patella length only after their death. Left and right front legs were removed and photographed under a microscope, and the tibia-patella length was measured with the measuring tool in the software *Leica Application Suite V4.6* (Leica Microsystems (Switzerland) Limited). We used the adult weight to assign females to three size classes prior to the mating trials. We later confirmed that adult weight is an acceptable approximation of body size as it correlated strongly with leg length (Spearman correlation: *N* = 119, rho = 0.952, *p* < 0.0001). As a measure of fecundity, we took the weight of the first egg sac from each female.

### Experimental set-up

Adult females were placed in open plastic frames (Fig. 1a) scratched from the inside to facilitate silk attachment and web building. The frames were fitted with two holes in the bottom to fix them on the floor of the choice arenas. Until the females participated in the tests, we kept each frame in a 3 l plastic box with air holes that were covered with gauze. The boxes were covered with petroleum jelly from the inside. That prevented females from walking around and allowed them only to build their webs in the frames. To encourage females to build webs, we put one *Calliphora* in each plastic box. Females were frozen at − 80 °C after they died a natural death in the laboratory, or after they had produced two egg sacs. Males either died during the mating trials by sexual cannibalism, or they died naturally in the laboratory. Dead males were also stored at − 80 °C.

**Fig. 3:**
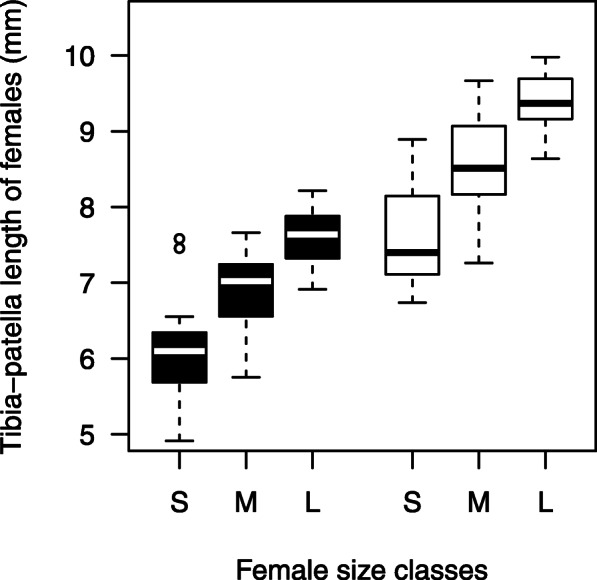
Summary of female size class conditions. The boxplots present the body size (leg length) differences in Northern (black) and Southern (white) females of different size classes (S = small, M = medium-sized, L = large)

We designed choice arenas (Fig. 1b) to resemble a natural situation for a mate-searching male closely. Males entered the arena by walking down a stick attached to a platform at one of the shorter sides of the arena. A fan on the opposite end secured a constant weak airflow ensuring that the male perceived volatile pheromones from the three females that resided in their webs inside the arena. Those three females either originated from the Northern or the Southern population only. Since females of both origins significantly differed in adult weight (Welch t-test: N_Northern_ = 63; N_Southern_ = 59, t = − 9.7078, df = 99.478 *p* < 0.0001) and leg length (t test: N_Northern_ = 61; N_Southern_ = 58, df = 117, t = − 11.422, *p* < 0.0001) we divided the three females into population-specific size classes (small, medium, large). The difference in adult weight between the small and large female to the medium one had to be at least 20 mg. A posteriori, we could confirm that in both treatments, the leg length of females differed significantly according to their size class (Fig. 3, linear regression: Northern females: *N* = 76, F_2,73_ = 63.71, *p* < 0.0001; Southern females: *N* = 80, F_2,77_ = 68.42, *p* < 0.0001). Moreover, pairwise comparisons revealed that between treatments, only “large” Northern females and “small” Southern females had a similar size (Pairwise t test: *N* = 51, *p* = 0.83), while in all other size classes, Southern females were larger (Pairwise t test: all comparisions revealed a maximum *p* < 0.0001). In each test, the three females were closely matched for post-maturation age and never differed in age for more than 2 days. Throughout the experiment, we used females that had a similar age that lay between four and 8 days (median = 5 days; interquartile range: 4–6 days). The positions of the three females in the arena were at different, fixed distances to the starting point (see Fig. 1b). By alternating the positions of the different size-classes, we achieved a balanced distribution of female size classes on all positions.

Males had 1 h to find the first female before we replaced them. We observed the search and the mating outcome of each male in real time. Once a male had found a female, we measured the copulation duration of the first mating with a stop watch, noted the occurrence of sexual cannibalism and determined his mating tactic: a male was defined as monogynous, if he copulated once and was cannibalised by the female or if he copulated twice with the same female. However, we only considered the latter option as a chosen mating tactic because we were unsure whether male death after the first copulation occurred willingly. A male was defined as bigynous, if he copulated once with the first female and then intended a bigynous tactic by leaving her web for at least one hour. In nature, it is extremely unlikely that males leave a female and then come back. Within this hour, we noted whether males visited and mated with one of the two other females. If males did not visit a second female, which was the case in 87% of the males, we still considered males as bigynous if they showed no intention to mate with the first female again. Conditions for the classification as intentionally bigynous were that these males had no contact to the web or the test frame of their previous mate anymore. We planned 60 trials in total, 30 for each female origin but had to exclude three trials from each subset. The post hoc exclusion was conducted as a precaution against potential biases due to developmental anomalies in a group of males. Since the excluded males were all cannibalised, they did not affect the main objective of the study (effects of female size on the probability of monogyny in surviving males).

We used six arenas in parallel, which were placed outdoors. Arenas were cleaned with ethanol between trials. To fulfil our conditions of age-matched females and minimal adult weight differences of 20 mg, we re-used some females but only those that had no prior contact with males. In total, we used 34 of the 122 females in more than one test, but never more than three times (mean 2.2). Re-used females were evenly distributed across both origins and all size classes so that potential biases can be excluded.

### Statistics

We used R 3.0.3 [73] for the statistical analyses. Unless otherwise specified, we stated the mean and the standard error of continuous data. We checked whether data were normally distributed and chose parametric or non-parametric tests accordingly. Most of the data was analysed with statistical models.

To test whether the copulation duration was affected by the females’ geographical origin (Northern, Southern) or female size class (small, medium, large), we applied a linear regression with both explanatory variables and simplified the model stepwise. We found two outliers that negatively affected the model fit. We present the results without these outliers, as the in- or exclusion did not change the significance of the models.

We used separate binary logistic models to test how female size affected the probability of cannibalism after the first copulation and the probability of monogyny and bigyny in surviving males. By restricting the latter analyses to males that survived their first copulation, we ensured that monogyny was an unambiguous male decision without potential influences of the females’ cannibalistic attacks during the first copulation. As explanatory variables, we used the females’ geographical origin, the female size class and their interaction. In previous models, we also included the adult age of males because it had a wide range between 8 and 34 days (20.7 ± 5.5 days) and could likely affect male decisions [74]. Since we found that male age was not informative, we decided to simplify the results and only present the effects of female quality.

Since our female size classes had no strict borders, we also dissolved the size classes and used female size (leg length) as a continuous variable on the probability of monogyny. We also explored the fitness outcome of monogynous and bigynous males. As an index for fitness, we used the number of eggs produced for the first egg sac. Counting eggs would have been destructive, which we had to avoid due to upcoming experiments. Instead, we used a mathematical equation to translate the weight of the first egg sac into an approximate number of produced eggs. To receive the function, we used laboratory data of 66 Northern females (see supplemental material) that did not participate in this study and of which we knew the weight of the egg sac plus how many eggs the egg sac contained. We found a strong positive relationship between weight and number of eggs per egg sac (linear regression: *N* = 66, F_1,64_ = 127.06, *p* < 0.0001, adjusted R^2^ = 0.6598). Therefore, we could use the estimates of the linear regression to create the mathematical equation (y = 1.3029x + 74.9097).

In logistic regressions, particularly with small sample sizes, a stepwise reduction is discussed to be an unreliable method [75]. Therefore, we applied a best model selection approach to all logistic regressions instead. We used the package “MuMIn” [76] and compared all possible models that could be constructed from the above mentioned female quality predictors. For the selection of the most plausible model, we calculated the Akaike information criterion corrected for small samples (AICc) of each model and accepted the model with the lowest AICc value as the most plausible model. If several models had a similar AICc value (difference of two units to the most plausible model), we considered them as equally plausible [77]. Eventually, we chose from these models the one with the fewest parameters, because any additional parameters would not be informative [77]. When we tested for the effect of the female size class on the probability of monogyny, we found that the standard errors of the estimates were very high (due to a quasi-complete separation). Therefore, we used the package “mbest” [78] and applied a Firth bias correction to the logistic regression.

## Supplementary information

**Additional file 1.** Raw data of mating tests. Table including the raw data of the experiment.

## Data Availability

All data generated or analysed during this study are included in this published article and its supplementary information files.

## Abbreviations

AICc: Akaike information criterion corrected for small samples
L: Large
M: Medium-sized
S: Small
SE: Standard error
